# Child death review: understanding variations in practice using normalisation process theory

**DOI:** 10.1136/bmjpo-2025-003432

**Published:** 2025-05-27

**Authors:** Karen Shaw, Sara Kenyon, Anna Pease, Jenna Spry, Gayle Routledge, Joanna Jane Garstang

**Affiliations:** 1 University of Birmingham College of Medical and Dental Sciences, Birmingham, England, UK; 2 Centre for Child and Adolescent Health, University of Bristol School of Social and Community Medicine, Bristol, UK; 3 University of Bristol, Bristol, England, UK; 4 A Child of Mine, Stafford, UK; 5 College of Medicine and Health, University of Birmingham, Birmingham, UK; 6 Birmingham Community Healthcare NHS Foundation Trust, Aston, UK

**Keywords:** Qualitative research, Mortality, Health services research

## Abstract

**Background:**

In England**,** Child Death Review (CDR) is a statutory process designed to identify full reasons for death, support parents, improve care and save lives. At CDR meetings, professionals review care and identify learning. Although parents do not attend CDR meetings, they should be invited to contribute questions and feedback and be informed of outcomes. There is a lack of evidence to support implementation, particularly for families after an expected child death.

**Objective:**

To examine how parent involvement in CDR is operationalised currently, following expected child death, to support improvements in practice.

**Methods:**

A secure online questionnaire was developed to collect data on parental CDR involvement in hospitals and palliative care services in England. Semistructured interviews were undertaken during 2022–2023, with 21 professionals in five care settings, purposively sampled from the survey to include sites with different provision contexts. Quantitative data were analysed using basic descriptive statistics. Qualitative data were analysed using directed qualitative content analysis and through a conceptual lens of Normalisation Process Theory.

**Results:**

Questionnaires were completed by 13 Paediatric Intensive Care Units and 16 palliative care services. 25/29 (86%) held CDR meetings reflecting statutory guidance, 17/29 (59%) routinely informed parents about CDR and 10/29 (28%) shared outcomes with them. Interviews with 21 professionals revealed that despite valuing CDR, many struggled to implement the process and lacked confidence to involve parents. Professionals felt that parents need good bereavement support to be able to contribute and wanted resources to help inform parents about CDR and support their involvement. Enthusiastic leaders were important. Lack of funding, particularly for CDR and bereavement keyworkers, was a challenge.

**Conclusions:**

**WHAT IS ALREADY KNOWN ON THIS TOPIC**

- Statutory guidance states that bereaved parents should be informed of Child Death Review, be able to give feedback for reviews and be told outcomes; but there is little practical information on how to do this.
- Parents have unique insights into their child’s treatment and care that could inform reviews.
- Many bereaved parents want to contribute to Child Death Review.

**WHAT THIS STUDY ADDS**

- Although most healthcare trusts hold Child Death Review meetings, often parents are not informed of these or able to contribute information.
- Professionals have limited confidence in involving parents in Child Death Review.
- The lack of a structured process limits professionals’ ability to support parents’ involvement in Child Death Review.

**HOW THIS STUDY MIGHT AFFECT RESEARCH, PRACTICE OR POLICY**

- This study has highlighted the need for funding and training for bereavement keyworkers to support families through the Child Death Review process
- Enabling parents’ involvement in Child Death Review should lead to greater learning from deaths and help improve future care.

## Background

 Childhood death is a reality for many families. Every year in the USA, approximately 38 000 children die before their 18th birthday,^1^ as do 13 000 in the European Union.^2^ In England alone, there are 3500 child deaths annually, with around one-third from acute or chronic medical conditions, malignancy, congenital abnormalities or genetic disease illness.^3^ Understanding the circumstances around a child’s death is important for parents, including in situations where death is anticipated.^4^ Sense-making is an important part of grieving,^5^ and lack of support around this time is associated with complex grief and increased rates of morbidity and mortality.^6 7^

Understanding childhood death is also important for service improvement. Indeed, Child Death Review (CDR) is an essential mechanism to improve paediatric healthcare, by identifying factors that could prevent future deaths and improve care.^8^ While this has historically focused on unexpected deaths, the importance of reviewing deaths following chronic illness or disability has been highlighted.^9^ This is reflected in CDR for England; a statutory process underpinned by operational guidance.^10^ This guidance sets out expectations that a multi-agency, holistic, CDR meeting (CDRM) for professionals is held several weeks after death, organised by the healthcare organisation caring for the child. Parents do not attend CDR meetings to enable open discussion among the range of professionals involved in their care.

Importantly, parents should be informed of the CDR process, given opportunities to feed into meetings and receive information about the outcomes. This involves allocating families a keyworker to signpost supportive services and act as a single point of contact between parents and the CDR process. However, while parent involvement is endorsed, there is limited practical information to support implementation. This is particularly true for anticipated deaths that occur in hospital or palliative care services, where guidance to support families lags behind that for perinatal^11^ or sudden unexpected deaths.^12^

Involving parents in CDR is important, as they may be uniquely aware of certain treatment or care issues.^13 14^ Unfortunately, hospital enquiries into avoidable infant and child deaths show clinicians have failed to listen to parents’ concerns, investigate deaths adequately and communicate effectively with families.^15^,^17^ There is an urgent need, therefore, to examine how to implement parent involvement in CDR in ways that support families, improve care and prevent future deaths. The current study has examined the factors that shape parent involvement, with a focus on how professionals work individually and collectively to implement recommended practices. This was part of a wider research project to co-design a best-practice toolkit for parental involvement in CDR; details of parent experiences (in press) and co-design elements^18^ have been reported separately.

## Methods

### Design

A sequential explanatory mixed-methods design was used, starting with an online survey of CDR professionals in England to explore current practices related to parent involvement and level of implementation. The results were used to purposively select five National Health Service (NHS) Trusts to participate in a qualitative study and explore the factors that shape implementation.

### Setting and eligibility criteria

Eligibility criteria included healthcare professionals based in English hospitals, hospices or community palliative care teams involved in CDR for children aged between 1 month and 18 years (post-neonatal child deaths).

### Survey

A secure online questionnaire was designed to collect data on: participant and organisational characteristics, CDR implementation and parental CDR involvement. It used non-randomised adaptive questioning and was pretested by three members of NHS staff.

Most inpatient child deaths occur in level three Paediatric Intensive Care Units (PICU). Invitations and survey links were therefore distributed to all 21 English Level 3 PICUs, via the Paediatric Critical Care Society (PCCS), requesting completion by the lead clinician responsible for mortality review.

Palliative care services that hold CDRMs were also eligible to participate. However, in the absence of a national list of relevant services, support was sought from all 58 Child Death Overview Panels (CDOP) to identify and invite lead clinicians responsible for CDRMs in their regions.

Surveys were distributed between November 2022 and March 2023. Peak winter pressures and the ethical requirement to send the survey through PCCS meant reminders were not sent to PICU non-responders, one reminder was sent to other non-responders.

### Interviews

Five NHS Trusts were purposively selected to participate in the interview study, chosen to reflect variation in geographical location, type of provider, number of yearly deaths and level of current implementation. Each site identified key staff related to CDR, who were invited to participate in semistructured interviews (online or telephone as preferred). These were undertaken by JS (female qualitative researcher with experience of palliative care studies and PICU nursing).

Question topics included their experiences, barriers and facilitators to involving parents in the CDR process. A distress protocol was developed, but use was not required. Informed consent was obtained, including permission to audio-record and transcribe interviews (verbatim and anonymised).

### Analysis

Standard descriptive statistics were used to analyse quantitative survey data. Qualitative data (surveys and interviews) were analysed using directed qualitative content analysis^19^ and managed using NVivo software (V.14).^20^ The transcripts were analysed by 3 members of the research team (JS, KS, JG) with a selection cross-checked by a fourth member (AP). The analysis approach was informed by Normalisation Process Theory (NPT).^21^ NPT proposes four constructs (with 16 subdimensions) to understand how complex interventions or new ways of working, like CRD, become embedded and routinised into clinical practice. The first two are ‘planning phases’ of work^22^ where individuals, as part of social groups, try to make sense of new practices (Coherence) and organise themselves around the ideas, objects and requirements of these (Cognitive Participation).^21^ The latter two are ‘doing phases’^22^ where people undertake the practices (Collective Action), evaluate them and take responsive action (Reflexive Monitoring).^21^ This is an established framework that has been used previously in related fields.^23 24^ Data interpretation was supported by regular discussion with the wider project team.

### Ethics

The project was approved by HRA and HCW (reference 22/WM/0172, 27 September 2022). The sponsor was the Birmingham Community Healthcare NHS Trust.

### Patient and public involvement

The study patient and public involvement and engagement advisory group consisted of bereaved parents and members of organisations and charities that support bereaved parents. This group was instrumental in the development of the protocol, interview schedule, distress protocol, selection of sites and data sense-checking.

## Results

### Survey responses

In total, 29 survey responses were received, including 13 from the clinical leads of Level 3 PICUs (response rate of 62%) and 16 from palliative care services who hold CDRMs. The response rate for palliative care can only be estimated, as the number of eligible services was unknown. However, the intention was to identify a lead palliative care clinician responsible for CDR in each CDOP region (n=58). Eligible contacts were provided by 38 CDOPS. Of these, 16 participated (suggesting a response rate of 28%), including lead CDR professionals in community/hospice settings (n=7) and district hospitals without a PICU (n=9).

### Levels of implementation

**Figure 1:**
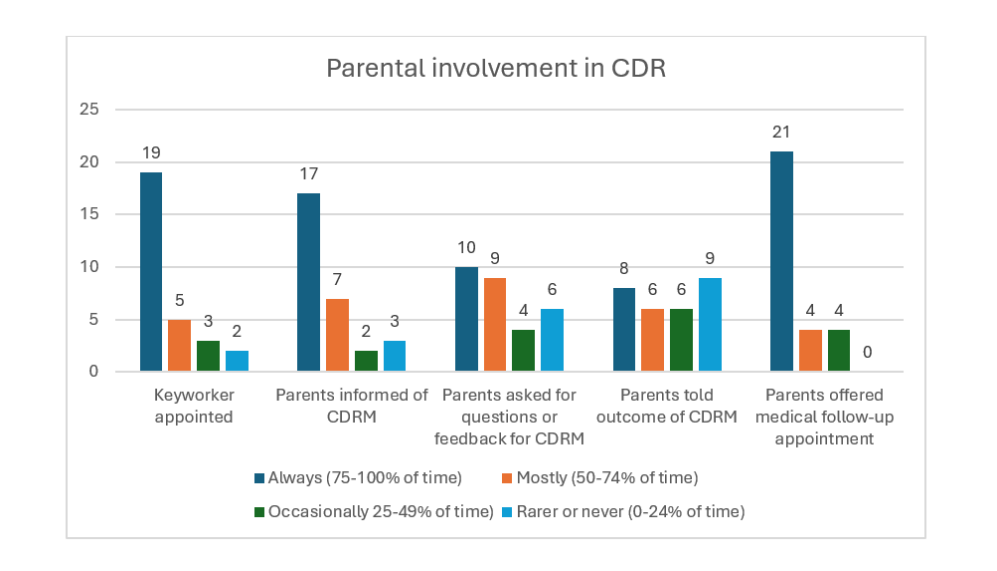
Parental involvement in Child Death Review (CDR).

Analysis of the survey data showed wide variation in CDR implementation, particularly in regards to parent involvement. Most (25/29, 86%) reported holding CDRMs in formats reflecting the statutory guidance (ie, one review meeting per child with all specialities represented). The remaining four services held separate review meetings for each specialty treating the child or had no review meeting. However, implementation of parental involvement was less evident. Just over half of services reported that parents were routinely assigned a keyworker or informed about CDR. Even fewer always gave parents an opportunity to input into CDR meetings (10/29, 34%) or receive feedback about the outcome (8/29, 28%). The variation in parental involvement in CDR is shown in figure 1.

### Interview study

To understand these reported variations in parent involvement, five respondent sites participated in a qualitative interview study; purposively selected to include different service contexts as shown in table 1.

**Table 1:** Details of sites for professional interviews

Cases	Deaths per year	Type of provider	Level of CDR implementation	Involvement of parents	Number of interviews
Site 1	Over 20	Hospital with PICU	Implemented	Routine involvement	5
Site 2	Over 20	Hospital with PICU	Partially implemented	Variable involvement	3
Site 3	Less than 20	Hospital with smaller PICU	Partially implemented	Variable involvement	2
Site 4	Less than 10	Community palliative care service	Implemented	Routine involvement	6
Site 5	Less than 10	Community palliative care service	Implemented	Not routinely involved	5

CDR, Child Death Review; PICU, Paediatric Intensive Care Units.

With permission, clinical leads shared contact details for 30 eligible individuals, 21 of whom consented to semi-structured interviews by video conference (n=20) or telephone (n=1). Reasons for non-participation included lack of availability and non-response, despite reminders. Details of interview participants are shown in table 2.

**Table 2:** Details of professionals interviewed

Job role	Number of interviewees
PICU consultant	5
Community paediatric or palliative care consultant	3
Senior or specialist nurse	6
CDR coordinators	4
Bereavement support workers	3
Total	21

CDR, Child Death Review; PICU, Paediatric Intensive Care Units.

Examination of the survey and interview data, through a lens of NPT, revealed many factors that impact on the potential for parent involvement to become routinised in CDR. These related to how professionals and services were (i) making sense of CDR, (ii) making it happen, (iii) working together and (iv) judging its value. Participant quotes relating to the different domains of NPT are shown in tables3^6^).

**Table 3:** Participant quotes relating to making sense of the CDR (Coherence)

Theme	Reference number	Quote
Different from previous working	1.1	I think 10 years ago, there wasn’t a statutory guidance that everyone had to do a Child Death Review. Now it’s very clear you have to, and there are some key points in that statutory guidance where you have to adhere to. And I think that’s helped, so at least that it doesn’t depend on postcode, where you were born or where you get into hospital, that at least there’s some form of review for every child that dies. (Consultant 1)
New focus on expected deaths	1.2	I think it runs much more smoothly when it’s an unexpected death and there is a joint agency response nurse who liaises with the family and will seek the feedback. I think the expected deaths - I think the families do miss out. (Consultant 2)
Importance of involving parents	1.3	While on PICU, what we see is mainly PICU bits and you might get an ambulance crew who are aware what happened at the roadside. The GP might know something about the context, but putting everything together, you kind of need the parents. (Consultant 1)
	1.4	It just gives that to the family, gives them that relief knowing that people care about what they’re saying, what they’re asking and that their child hasn’t been forgotten, they’re not just a number. (Nurse 1)
Importance of bereavement support	1.5	I think that relationship and that inherent trust is important, so it’s not actually just about the CDR process. It’s actually about the entirety of the bereavement and grief process, and how do we support that? In order for them (parents) to ask questions and get involved, they have to have a trust and a relationship to that. So it’s the support around it which actually we need to look at rather than their direct involvement in CDR (Consultant 3)
Misunderstanding CDR	1.6	We do the M&M (morbidity and mortality) meetings which I think’s probably another name for the Child Death Review meeting as opposed to the panel that’s off site that meets regionally. So, I suppose we just call it something different. (Nurse 2)
Wrong information given to parents	1.7	I had one family who were given the wrong information (or parents misunderstood the information) and thought that the CDRM was a sort of inquest. This caused a lot of upset when the family realised they weren't able to attend. Getting it right at the start with an appropriately trained key worker, I feel, would have prevented this distress. (Consultant 4)
Scepticism about CDR	1.8	These days, everyone’s desperate, aren’t they, for us to find things to learn from…we mostly know…to improve things, you need more experienced staff, you need more resources, etc., and a lot of that doesn’t seem to change. (Consultant 1)

CDR, Child Death Review.

**Table 4:** Participant quotes relating to making it happen (cognitive participation)

Theme	Reference number	Quote
Parental involvement as core element of CDR	2.1	I feel very secure, in that I believe our Child Death Reviews are done well and thoroughly and that they give the child and the family the sort of time and dignity and respect that they need. (Nurse 3)
Coordination across services	2.2	We did a lot with the medical examiner…we’re trying to make sure our links with the coroners are stronger…We have a paediatric palliative care network meetings and we certainly have had training talks at them. (Consultant 2)
Service led changes	2.3	We’re in the process of doing our End-of-Life Care at Home policy, which incorporates our Expected Death policy which talks about CDRM and CDOP [Child Death Overview Panel]. (Nurse 4)
Lack of funding	2.4	But one of the reasons why the trust hasn’t done the CDRM process probably is because there’s no funding from it within the hospital, so there’s no one to do it, there’s no time to really do it. (Consultant 2)
Not prioritising parents’ involvement in CDR	2.5	Although parents are aware there will be a review meeting, we have not actively asked for their participation in anything they would want shared or discussed. We also do not routinely tell parents the outcome. Maybe this is because we don't want to distress them or maybe because we are not the keyworker. (CDR lead – survey comment)

CDR, Child Death Review; CDRM, CDR meeting.

**Table 5:** Participant quotes relating to working together to involve parents (collective action)

Theme	Reference number	Quote
Importance of bereavement support teams	3.1	I think for me, the crucial part of the service are the bereavement and support team, so our palliative care and bereavement and support team. They’re absolutely brilliant. This all would fall apart if you wouldn’t have these nurses. (Consultant 3)
Need for individualised approaches	3.2	Even within one family, different parents might have different needs. So there might be a father that’s asking lots of questions and mum isn’t, and you need to be able to meet the needs of each parent and the family as a whole. (Nurse 3)
Building trust and empowering parents	3.3	I always say ‘I’m your voice, I’m your child’s voice at that meeting. If there’s anything you want me to bring up, let me know’.(Nurse 5)
Timing and pacing of conversations	3.4	So, on the first phone call I’ll say, ‘do you want me to give you another ring this week?’ and most of them say ‘yes’ and if they say no I’ll say, ‘I’ll give you another call next week, but I’ll text you first just to make sure it’s okay to ring’ or I’ll say, ‘do you want me to ring you or do you want to ring me when you want support?’(Nurse 5)
Keyworkers not understanding their role in CDR	3.5	We don’t have any bereavement nurses in our trust…,.I have tried to use those staff that know the family better to ask the family for feedback prior to the meeting. That doesn’t always work that well because I think the staff don’t …necessarily understand the process. (Consultant 4)
Importance of early discussions	3.6	In patients where there have been early discussions about child death, there can be better engagement in the end of life and post-death processes. When discussions are left late, it can be difficult to facilitate important conversations after the event”(CDR lead – survey comment)
Lack of resource	3.7	My role is purely the Child Death Review coordinator and it always has been. From our perspective, yeah, we could do with someone here at least full time, and I do work a lot of hours unpaid. (CDR coordinator 1)
	3.8	Now, normally we just do work for free, as slightly gullible young consultants, and this time we made a point that we cannot, we’re not qualified to look at the nursing care, without a nurse…if you insist on clinicians coming, you have to probably give clinician time, to make it work. (Consultant 5)
Lack of training	3.9	It was very much, ‘Off you go,’ and kind of left on my own, so it’s been very much make the role my own, at my level, and do my best. So learning as I go (Bereavement support worker 1)
Missed opportunities	3.10	We now have a system for organising and managing CDRMs and parents are invited to give feedback/questions. Some of these children die outside of the Trust as they have accessed a specialty service, for example, oncology. I believe the families are asked for their input, but not entirely sure, and I am not always invited the to CDRM. (lead CDR nurse – survey response)

CDR, Child Death Review; CDRM, Child Death Review meeting.

**Table 6:** Participant quotes relating to understanding the value of CDR (reflexive monitoring)

Theme	Reference number	Quote
Raising awareness of CDR	4.1	If I were to turn around to a community nurse tomorrow and say, ‘Have you heard of CDRM?’ they’d say, ‘What?’ They don’t know and actually, a lot of our kids get put through a CDRM and they need to be a part of it. They panic about it. I had someone the other day with anxiety about going, and I said, ‘What do you think is going to happen?’…I said, ‘No, if you’ve done something wrong, you’d know about it by now’. They panic because it brings up a lot of emotions for them. (Nurse 4)
Need to monitor the impact of CDRM	4.2	People are coming up with these learning points…but then often there’s no process for seeing if anything ever actually changed afterwards. (Consultant 6)
Challenge of getting feedback from parents	4.3	I think it’s fair to say it’s a challenge to get feedback and I don’t think we have actually mastered how best to do it. (Consultant 2)

CDR, Child Death Review.

### Making sense of Child Death Review (CDR) (Coherence)

Most participants welcomed CDR as key to improving standards in care; distinguishing it positively from previous working (quote 1.1). Some valued its increased focus on reviewing expected deaths, explaining how processes lagged behind those for unexpected deaths (quote 1.2). However, many struggled to implement statutory guidance and expressed concern about the ‘difference in quality’ between regions and organisations. Despite an explicit agenda to discuss parent involvement, the concerns of many professionals were more broadly focused on how to make the CDR itself work effectively. They were unclear about the optimal approach and found it challenging to include parents in a process they lacked confidence in. This reflects the NPT assertion that successful implementation requires that people can make sense of it and its associated practice.

That said, there were many shared beliefs. CDR was viewed as an ‘educational’ process, ‘for staff’ to improve care and reduce preventable deaths. Parental involvement provided ‘invaluable’ insights from experience and unique oversight of the ‘whole process’ of care (quote 1.3). Parents could provide insights about the value of care; acknowledging ‘what they perceive as useful might be entirely different*’* to service providers. Parental input helped centre CDRMs on the needs of children and families, rather than personal agendas. Their input promoted multidisciplinary working, aligning discussions on ‘the same goal’ to ‘answer questions related to the child’s care’.

Parental involvement also reflected broader existential and democratic principles in healthcare. The CDR offered an important ‘platform’ where parents and professionals could work together to ‘honour a child’s death’. Parents were seen as an ‘advocate’ or ‘surrogate marker of that child’s voice’ and had a ‘right’ to be involved in discussions about their child. Enacting these principles through CDR was felt to support humanised care, parental well-being and staff satisfaction (quote 1.4).

As parents do not attend CDRMs in person, this enabled ‘open discussions’, without professionals worrying about parental distress or having to support their understanding. However, they stressed transparency, advocating for ‘no secrets’ and ‘honest’ feedback. They urged professionals to ‘empower’ parents to be involved, and not act as ‘gatekeepers’. Involvement should be an ‘informed choice’, recognising that not all parents benefit.

Despite these benefits, CDR was widely experienced as ‘complex’, ‘messy’ and ‘time-consuming’ requiring the interplay of many professionals. The quality of parent involvement depended on the quality of bereavement support (quote 1.5). Complexity varied, ranging from a ‘straightforward process’ where families are well known to professionals, to challenges of involving parents with whom they had no existing relationship or where post-mortem examinations or coroners were involved.

Existing statutory guidance^10^ did not enable professionals to navigate this complexity. Not all professionals were clear that CDR is statutory, and some had differential interpretations of what was expected. Boundaries between CDR and other operational processes or purposes were not always clear, distinct or understood. Some wrongly believed parent involvement was limited to specific cases (eg, ‘complex patients’, parents with complaints).

Others mistakenly equated single-specialty medical reviews with CDRMs, contradicting guidance (quote 1.6).

Lack of understanding or trust in CDR reduced professionals’ confidence to involve parents, and they expressed fears of exacerbating distress, sometimes validated by experience (quote 1.7).

A minority were sceptical about the benefits of changing existing review mechanisms, questioning whether new processes justified the costs and risks, especially given under-resourcing in healthcare (quote 1.8). It was evident that while professionals value review processes, their commitment to involve parents in the CDR was shaped by their understanding of CDR itself.

### Making it happen (cognitive participation)

NPT suggests that new ways of working require work to develop and sustain practices. Implementation of CDR relied on networks of enthusiasts interpreting and applying statutory guidance locally. This included regional clinical groups, national experts and service-level staff with special interests. They have integrated CDR into existing pathways, developed resources, supported training, created roles, facilitated inter-agency relationships and tested new ways of working, including parental involvement. However, this approach led to the varied understandings and commitments to CDR described, shaped by funding, leadership and local practices. Some sites described parental involvement as a core element of CDR, underpinned by sustained efforts to build practices that ensure parents are routinely involved (quote 2.1). They explained how this involved ongoing work, co-ordinated by regional clinical networks, to identify key people across relevant organisations and engage them to develop a unified approach (quote 2.2). There were also several examples of service-led changes to clarify expectations (quote 2.3).

Others struggled to reorganise existing ways of working to involve parents, particularly where deaths were high or leadership was lacking. Lack of funding (quote 2.4) led to compromises such as ‘CDRM lite’ or remaining with established review mechanisms, where parent involvement was not a priority.

### Working together to involve parents (collective action)

Participants believed many disciplines and organisations contribute to CDR. They emphasised the role of palliative care or bereavement teams in supporting parents’ involvement in CDR (quote 3.1). Discussions centred on the skill sets required to ‘tailor’ a ‘formal’ administrative and operational process to parents’ individual needs and circumstances, in a context of high distress and uncertainty about meeting timings. Keyworkers described how initial contact had to be timely but unfold in a ‘holistic’ and ‘bespoke’ manner, rather than being ‘prescriptive’. They described how they had to be sensitive to the family context (eg, ‘a quiet family’, ‘young couples’), aware of cultural issues, ‘never make assumptions’ and inclusive of wider family members (quote 3.2).

Parent trauma was seen as a major barrier. Participants felt that involvement in the CDR could cause the ‘re-living’ or ‘re-experience’ of trauma, so for them, it was understandable that parents may be resistant to being involved. It was also felt that some parents may find it less distressing to ask questions via a keyworker, rather than return to the place of their child’s death. Keyworkers needed a range of soft skills to ‘encourage’ families to engage with the CDR through relationships that were ‘honest’, ‘kind’ and ‘gentle’. They also describe the importance of framing information in specific ways to build trust and empower parents (quote 3.3). This included creating a permissive environment for them to discuss the ‘good and bad’, by explaining that professionals want to ‘uncover problems’, but also ‘what worked well for them’.

A key challenge was ‘holding’ parents during the process, which could be lengthy with no fixed timescale and shifting meeting dates. This required multiple contacts before, during and after CDR meetings. Keyworkers stressed the importance of timing and pacing of conversations, reassurance and optimising parents’ control (quote 3.4). Thus, the keyworker role was seen as pivotal, but often overlooked, with role allocation based on interest or alignment to existing duties (eg, bereavement nurses). However, sometimes the ‘best person’ may be other members of staff who know the family better but are not equipped to undertake the role (quote 3.5). Participants also stressed that identifying keyworkers and discussing the CDR was often done ‘ad hoc’ after death but could be planned earlier, given deaths were expected (quote 3.6).

Other key actors included the CDR Chairs who could demonstrate a commitment ‘to every child being given a good review’. These could signal the value of parent involvement and support meaningful feedback to parents. The crucial role of co-ordinators was also highlighted. Participants described how well-organised meetings made it easier to engage parents. However, some said this role was not always well understood or respected, which could undermine the process. While some sites legitimised CDR work by creating specific posts, most professionals undertook this work by absorbing it into their existing roles, making it challenging (quote 3.7). Participants described this as precarious, with people often seen as skilled and ‘motivated’, but ‘stretched beyond their capacity’. Being dependent on a small number of special interest professionals meant that the CDR process could ‘fall apart’ and get ‘behind’ when staff were away from work or had left the post. Lack of funding also meant that many professionals did ‘work for free’ (quote 3.8).

Participants also highlighted the lack of uniform job descriptions and formal training in CDR. In response, many had tried to fill the gap through local training, or peer support through buddying or mentoring. However, not everyone had the benefit of this (quote 3.9). The lack of role clarity and structured responsibility-sharing led to missed opportunities for involvement (quote 3.10).

### Understanding the value of Child Death Review (CDR) (reflexive monitoring)

Participants felt CDR was part of a longer-term process of change and described their approaches as evolving, rather than finalised. Most were unclear on the optimal model and called for increased clarity about implementation, including parent involvement. Their appraisals were largely experiential, though many described how these reflective practices did support development. Some had also undertaken service-level assessments such as audits. However, they did call for more formal evidence to guide future working, including more support to raise awareness about the purpose of CDR and their role in the process (quote 4.1).

Participants sought more unified processes across settings, describing how different organisations and regions wanted things ‘done in a slightly different way’, which could be confusing and time-consuming. Increased data was needed to refine funding models, assess parent experiences, and evaluate impacts on care and outcomes (quote 4.2).

Participants highlighted unmet support needs, calling for help to close the theory-practice gap, training, clearer roles, practice-based guidelines, resources for parents, tools to benchmark practice and quality indicators.

While some parent resources existed, they felt these did not fully support engagement. They wondered if there was ‘a way of guiding them to questions’ and providing information about the CDR that did not ‘overload them with information’ or ‘undue stress’ (quote 4.3).

## Discussion

This study surveyed 29 professionals and interviewed 21 involved in CDR. Nearly all (25/29, 86%) services had adopted the principle of one review meeting per child, 17/29 (59%) reported always informing parents of CDR, but only 10/29 (34%) always invited parents to contribute. Interviews revealed professionals valued CDR but struggled to implement it and lacked confidence to involve parents. Professionals appreciate the potential for greater learning if parents contribute to CDR but felt that parents are only able to contribute with good bereavement support in place. Involving parents in CDR requires professionals to adopt new ways of working, and this requires additional resources which is challenging. Keyworkers play a vital role in supporting families; this role also needs funding. Professionals also wanted resources to help inform parents about CDR and support their involvement.

A strength of this study was the diversity of professionals interviewed, recruited from healthcare organisations with a range of CDR practices and differing implementation of the statutory guidance. This enabled us to learn from those organisations where parental involvement in CDR has been embedded for some time and identify the challenges for those struggling to embed CDR. Many of our findings from the professionals’ interviews concurred with our analysis of parents’ interviews (in press); both highlighted the importance of good bereavement support and keyworkers, and the need for bespoke resources to support parents’ involvement and feedback. We had a robust method of analysis, supported by Normalisation Process Theory, which offered a framework to position our results in a way that may be widely relevant to CDR. While based within the English CDR system, our findings could be used to help develop and implement parental involvement in CDR internationally, as many countries have CDR systems, although few have guidance for family involvement. Although the survey had a low response rate, it was completed by a range of organisations, allowing a breadth of practice to be captured. It is possible that organisations with less robust CDR practices would be less likely to participate in the survey, leading to an over-estimation of current parental involvement in CDR. Nonetheless, this study highlights existing expertise and suggests that wider professional engagement could help normalise parental involvement in CDR.

There is limited research on professionals’ experiences of involving parents in CDR to compare with our findings. A small study of English Child Death Overview Panels reported the lack of involvement of parents in CDR prior to the 2018 guidance.^25^ A systematic review reported staff feeling undertrained and ill-prepared for follow-up conversations with bereaved parents.^26^ A large US study of PICU deaths^27^ showed the importance for physicians of offering a follow-up consultation to bereaved families, in that by understanding parents’ perspectives they may improve future care. They identified further barriers of lack of organisational support for these appointments and resistance from some doctors in offering appointments. The study team then developed guidelines for follow-up appointments, highlighting the need for institutional support, asking parents for their questions prior to meetings, and when necessary holding multidisciplinary review meetings to address these questions before feeding back to families.^28^ These findings resonate closely with ours; however, our study shows the potential benefits and challenges for expected deaths occurring for expected child deaths across palliative care settings and PICU.

Parent involvement in CDR has largely developed through the commitment and goodwill of enthusiasts, developing informal cultures of good practice. Implementation varies by local context, with some lacking the resources, incentives or accountability to encourage routine implementation. Insufficient service resources are a major barrier, particularly a skilled workforce with sufficient time to support parent involvement. Transforming the workforce requires investment to address current shortages in medical, nursing and specialist staff to support families. Our findings suggest that important steps include clarifying goals and intended outcomes for parent involvement in CDR, and identifying the most effective practice and service models to achieve these. This will include understanding what local variation is appropriate for certain contexts and what is inappropriate, potentially leading to health inequalities. CDR requires adequate resources to function effectively, a challenge given current funding constraints and clinical pressures. However, the lack of effective CDR has been highlighted in the recent Thirlwell Inquiry, and a CDR statutory assurance framework will soon be published to hold NHS services accountable for CDR. Professionals also require practical resources to unify understanding and approaches, including information to clarify and support critical roles, especially keyworkers. These are addressed by the co-designed toolkit to support parental involvement in CDR, available at https://www.ncmd.info/guidance/parents-cdr-toolkit/. Finally, there is the potential for overlap with the newly introduced medical examiner system for all deaths; this requires relatives to be routinely asked if they have any concerns about care.^29^ However, a survey of PICU reported that most medical examiners delegate this responsibility to bereavement support staff or keyworkers.^30^

It will be important to evaluate parents’ involvement in CDR as this becomes normalised practice. We need to ensure that parents’ needs remain at the forefront; and we should seek to show the additional learning that comes from parents’ involvement in CDR. Child health professionals should be advocates for bereaved families, championing resources so that CDR can operate effectively. We hope to inspire child health professionals globally to support bereaved parents in shaping learning from and preventing future child deaths.

## Data Availability

Data are available upon reasonable request.
