# Supplementary material for: Child death review: understanding variations in practice using normalisation process theory
Source: BMJ Paediatr Open. 2025 May 27;9(1):e003432. doi: 10.1136/bmjpo-2025-003432 (PMC12121585; doi:10.1136/bmjpo-2025-003432)
Supplement: Supplementary file 1 [file bmjpo-2025-003432.reviewer_comments.pdf]

PEER REVIEW HISTORY

BMJ Paediatrics Open publishes all reviews undertaken for accepted manuscripts. Reviewers are asked to complete a checklist review form and are provided with free text boxes to elaborate on their assessment. These free text comments are reproduced below.

ARTICLE DETAILS

**TITLE (PROVISIONAL)**	Child Death Review; understanding variations in practice using Normalisation Process Theory.
**AUTHORS**	Shaw, Karen; Kenyon, Sara; Pease, Anna; Spry, Jenna; Routledge, Gayle; Garstang, Joanna

VERSION 1 - REVIEW

**REVIEWER NAME**	***Ian Mitchell***
**REVIEWER AFFILIATION**	University of Calgary
**REVIEWER CONFLICT OF INTEREST**	N/A
**AI DISCLOSURE**	No
**DATE REVIEW RETURNED**	27-Mar-2025

**GENERAL COMMENTS**	No comments. Clear objectives, well planned study, conclusions spelt out in detail, with supportive evidence. Suggestions for improvement in parental involvement. Throughout compassion for bereaved parents clear.

**REVIEWER NAME**	***Christy Norwood***
**REVIEWER AFFILIATION**	Sydney Children's Hospitals Network Randwick, Community Child Health
**REVIEWER CONFLICT OF INTEREST**	N/A
**AI DISCLOSURE**	No
**DATE REVIEW RETURNED**	11-Apr-2025

**GENERAL COMMENTS**	Great Article, interesting data collected with sensible and helpful recommendations.<br>Minor changes recommended<br>Page 1<br>Line 11<br>First sentence too long and doesn't read well. ? add a full stop after "saves lives" ie "saves lives. At CDR"<br>Background In England, Child Death Review (CDR) is a statutory process designed to identify full reasons for death, support parents, improve care and save lives, at CDR meetings professionals review care and identify learning<br><br>Page 2<br>line 22 doesn't quite make sense<br>Suggest Delete 'in' after operationalised?<br>Objective To examine how parent involvement in CDR is operationalised in currently, following expected child death, to support improvements in practice,

	Line 40 Change reflecting to reflected or reflect<br>Results Questionnaires were completed by 13 Paediatric Intensive Care Units (PICU) and 16 palliative care services. 25/29 (86%) held CDR meetings that reflecting statutory guidance, 17/29 (59%) routinely informed parents about CDR and 10/29 (28%) shared outcomes with them.<br><br>Page 8<br>Table 3<br>It would be good if you could make the different domains of NPT a little clearer. Ie separate the “Coherence”, “Cognitive Participation” etc sections. It is easy to get lost in the table<br><br>Page 17<br>Line 3<br>Missing a word?<br>Others struggled (?with) existing ways of working to involve parents, particularly where deaths were high or leadership was lacking<br><br>Page 19<br>Line 23 Missing a word?<br>, which offered a framework to position our results in (?a) way that may be widely relevant to CDR<br><br>Page 20<br>Line 13 add a comma after inappropriate<br>This will include understanding what local variation is appropriate for certain contexts and what is inappropriate, potentially leading to health inequalities.

VERSION 1 – AUTHOR RESPONSE

Reviewer comment	Changes
Page 1 Line 11<br>First sentence too long and doesn’t read well. ? add a full stop after “saves lives” ie “saves lives. At CDR”	In England, Child Death Review (CDR) is a statutory process designed to identify full reasons for death, support parents, improve care and save lives. At CDR meetings professionals review care and identify learning.
Page 2 line 22 doesn't quite make sense<br><br>Suggest Delete ‘in’ after operationalised?	Objective To examine how parent involvement in CDR is operationalised currently, following expected child death, to support improvements in practice.
Page 2 Line 40 Change reflecting to reflected or reflect	25/29 (86%) held CDR meetings reflecting statutory guidance,
Table 3<br><br>It would be good if you could make the different domains of NPT a little clearer. Ie separate the “Coherence”, “Cognitive Participation” etc sections. It is easy to get lost in the table	This has been separated into 4 different tables, one for each domain, presented with the narrative for each domain

Page 17 line 3<br><br>Missing a word?<br><br>Others struggled (?with) existing ways of working to involve parents, particularly where deaths were high or leadership was lacking	Others struggled with existing ways of working
Page 19<br>Line 23 Missing a word?<br>, which offered a framework to position our results in (?a) way that may be widely relevant to CDR	which offered a framework to position our results in a way that
Page 20<br>Line 13 add a comma after inappropriate<br>This will include understanding what local variation is appropriate for certain contexts and what is inappropriate, potentially leading to health inequalities.	This will include understanding what local variation is appropriate for certain contexts and what is inappropriate, potentially leading to health inequalities.

VERSION 2 - REVIEW

**REVIEWER NAME**	***Ian Mitchell***
**REVIEWER AFFILIATION**	University of Calgary
**REVIEWER CONFLICT OF INTEREST**	N/A
**AI DISCLOSURE**	No
**DATE REVIEW RETURNED**	12-May-2025

**GENERAL COMMENTS**	No comments, an important topic of parental involvement in child death review. this is clearly evolving and supports for parents also mean supports and training for staff. Likely funding also required

**REVIEWER NAME**	***Christy Norwood***
**REVIEWER AFFILIATION**	Sydney Children's Hospitals Network Randwick, Community Child Health
**REVIEWER CONFLICT OF INTEREST**	N/A
**AI DISCLOSURE**	No
**DATE REVIEW RETURNED**	11-May-2025

**GENERAL COMMENTS**	Looks great.
